# Beyond Atrial Fibrillation: Prioritizing Stroke Evaluation in Resource-Limited Settings

**DOI:** 10.7759/cureus.83774

**Published:** 2025-05-09

**Authors:** Hong Yee Lim, Yew Chung Chan, Wee Lee Chin

**Affiliations:** 1 Department of Internal Medicine, Tengku Ampuan Rahimah Hospital, Klang, MYS; 2 Department of Internal Medicine, Duchess of Kent Hospital, Sandakan, MYS; 3 Department of Cardiology, Raja Permaisuri Bainun Hospital, Ipoh, MYS

**Keywords:** adult congenital heart disease, atrial fibrillation, atrial septal defect, cardioembolic stroke, crochetage sign, electrocardiography

## Abstract

Atrial fibrillation (AF) accounts for the majority of cardioembolic strokes. In addition to AF screening, echocardiography is recommended to evaluate structural heart diseases including congenital anomalies. In resource-limited settings, in-hospital echocardiography for all stroke patients may not be feasible. However, careful assessment of readily available clinical parameters, such as bedside examination, electrocardiography, and chest X-ray, can offer essential clues to guide prioritization of echocardiography in selected patients and facilitate timely management. We present a case of recurrent cardioembolic stroke in a patient with AF, who was later found to have an underlying atrial septal defect, highlighting the importance of a comprehensive approach beyond AF in stroke evaluation.

## Introduction

Atrial fibrillation (AF) is one of the most common arrhythmias worldwide and a major cause of cardioembolic stroke. The prevalence of AF increases with age, reaching a peak of 5% in people over 65 years of age, and its incidence and prevalence are increasing [1]. Standard evaluation of cardioembolic stroke typically involves screening for AF and performing echocardiography to assess for structural heart disease [2,3]. However, in resource-limited settings, echocardiography is often delayed, particularly when AF is presumed to be the sole etiology. In such contexts, clinical examinations such as electrocardiography (ECG) and chest X-ray can offer valuable insights that can help prioritize early echocardiographic assessment.

## Case presentation

A 67-year-old man presented with a one-day history of right-sided weakness, facial asymmetry, and aphasia. Five months earlier, he had experienced an acute infarct involving the left temporo-occipital lobes, during which AF was detected. He was started on oral apixaban 5mg twice a day for stroke prevention. However, due to limited resources and a presumption that AF was the sole cause of the stroke, echocardiography was scheduled at a much later date. He had a 40-pack-year smoking history but had quit following his initial stroke. His past medical history was otherwise unremarkable.

**Figure 1:**
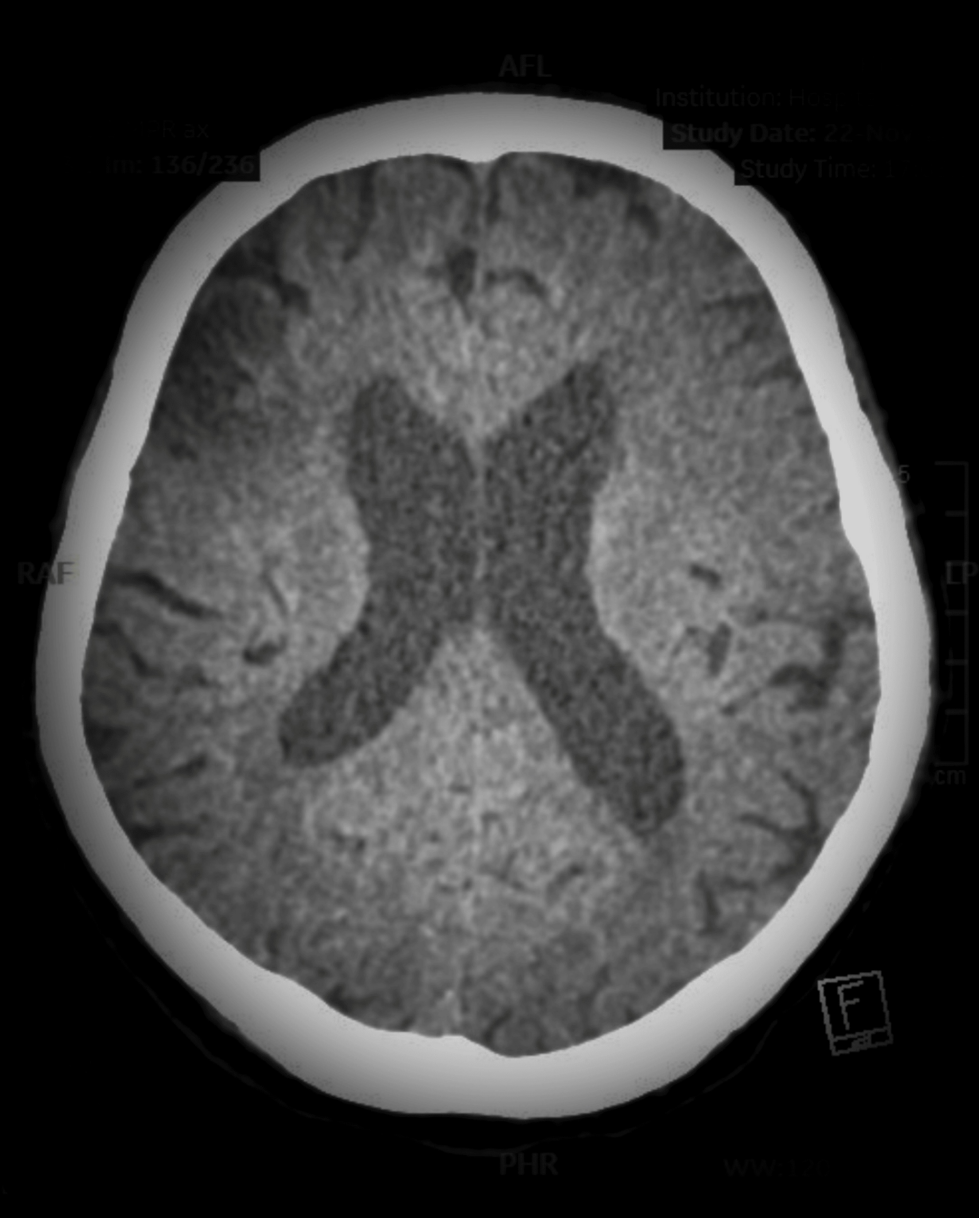
CT of the brain CT of the brain revealed acute infarct in the right middle cerebral artery territory, along with encephalomalacia in the right frontal and left temporo-occipital lobes. CT, computed tomography

On examination, he had a Glasgow Coma Scale (GCS) score of 11, with expressive aphasia, right-sided upper motor neuron facial palsy, and right-sided hemiplegia. Sensory examination was normal, and the other cranial nerves were intact. His blood pressure was 145/90 mmHg, heart rate was 80 beats per minute (bpm), and oxygen saturation was 100% on room air. Cardiovascular examination revealed an irregularly irregular pulse and a loud pulmonary component of the second heart sound (P2). There was no cardiac murmur, additional heart sound, or carotid bruit. A computed tomography (CT) of the brain revealed an acute infarct in the right middle cerebral artery territory, along with encephalomalacia in the right frontal and left temporo-occipital lobes (Figure 1).

**Figure 2:**
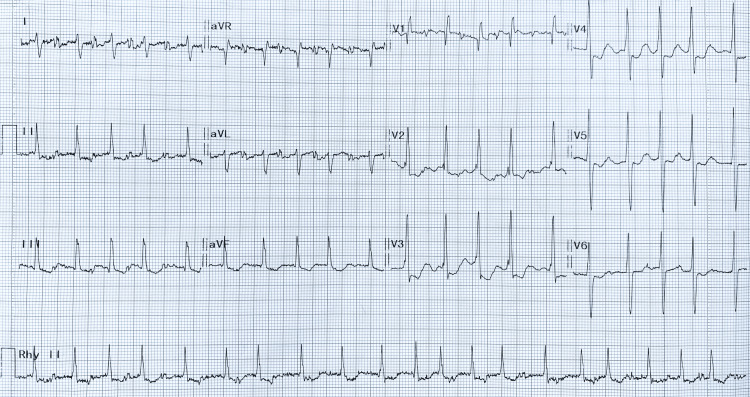
Electrocardiogram of the patient AF is noted by the absence of P waves and irregularly irregular QRS complexes. A positive QRS complex in lead aVF and negative QRS in lead I indicate right axis deviation. The presence of an rsR' pattern with a narrow QRS complex in lead V1 suggests partial RBBB. The crochetage sign is highlighted in the amplified ECG shown in Figure 3. RBBB, right bundle branch block

**Figure 3:**
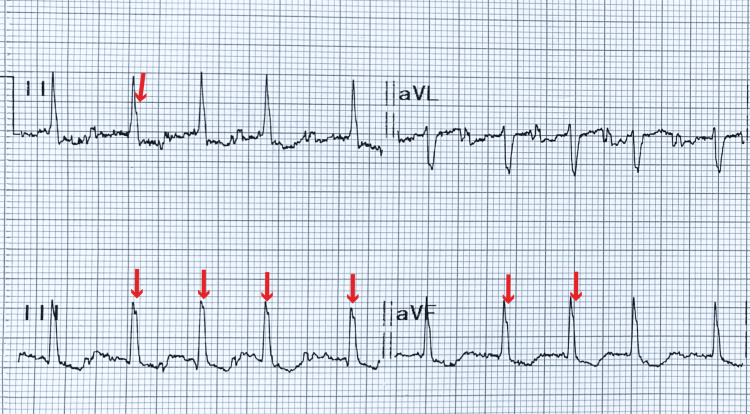
Amplified electrocardiogram of the inferior leads The crochetage sign is represented by a notching over the apex of the R wave in the inferior leads, as indicated by the red arrow. This sign is suggestive of an atrial septal defect.

**Figure 4:**
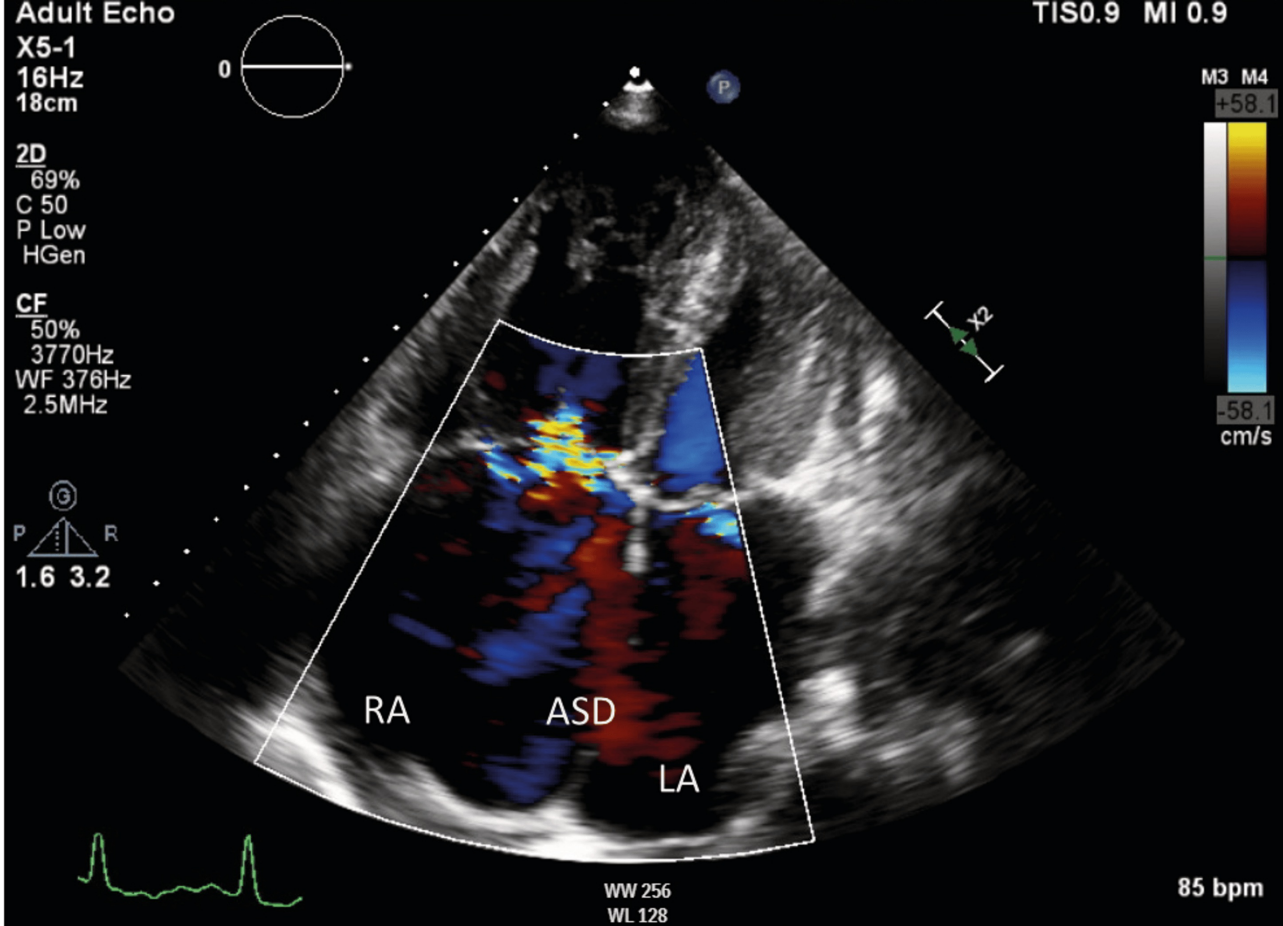
Apical four-chamber echocardiographic view The A4C echocardiogram demonstrates the presence of an ASD with a left-to-right shunt. Both the right and left atria appear dilated. ASD, atrial septal defect; LA, left atrium; RA, right atrium

Beyond AF, ECG demonstrated right axis deviation, partial right bundle branch block (RBBB), and a subtle notch at the apex of the R wave in leads II, III and aVF (crochetage sign) - a marker suggestive of atrial septal defect (ASD), an important yet under-recognized stroke risk (Figures 2, 3). Chest X-ray showed cardiomegaly. Echocardiography subsequently confirmed the presence of an ASD with left-to-right shunt and features of pulmonary hypertension (Figure 4). The patient was referred to a cardiologist for right heart catheterization and ASD closure. He was also scheduled for an outpatient carotid artery ultrasound examination as a part of stroke screening to rule out significant carotid artery disease.

## Discussion

AF is one of the most common arrhythmias worldwide and a major contributor to cardioembolic stroke. Current recommendations in cardioembolic stroke include screening for AF and performing echocardiography to assess for structural heart disease [2,3]. In many cases, the detection of AF in a stroke patient leads to a presumptive diagnosis of AF as the sole source of cardioembolic stroke. This pragmatic approach, however, often delays further cardiac evaluation. The term “lone AF” should no longer be used, as AF is often associated with a wide range of risk factors and comorbid conditions, including genetic predisposition, aging, ethnicity, gender, lifestyle-related factors, metabolic diseases, and structural heart abnormalities such as valvular defects and congenital heart diseases [2].

In addition to structural heart diseases, underlying coronary artery disease should also be considered and ruled out in patients initially presumed to have "lone AF." Recent studies indicate that up to 70% of AF patients may have coexisting coronary artery disease, which itself is an independent predictor of stroke [4-6]. This underscores the importance of comprehensive cardiovascular evaluation, particularly in high-risk individuals, rather than focusing solely on rhythm disturbances.

In developing countries with limited resources, echocardiography is not always readily available, especially in rural hospitals. As a result, when AF is detected and presumed to be the cause of cardioembolic stroke, echocardiography is often scheduled at a later time as part of routine screening. This is particularly true when the majority of stroke patients are expected to have normal echocardiographic findings [7]. Our case demonstrates the need for clinical risk stratification to prioritize urgent echocardiographic assessment in selected patients in order to prevent delayed diagnoses of structural heart diseases that require prompt treatment.

Congenital heart diseases, particularly left-to-right shunts such as ASD and ventricular septal defects, are often missed until later stages, especially in resource-limited settings. These conditions are major contributors to early onset heart failure and arrhythmias, including AF, in young adults. Early detection and targeted screening are essential to prevent irreversible complications and reduce the long-term burden of heart failure and stroke [8]. Relying solely on anticoagulant therapy in AF without addressing the underlying structural abnormalities may not adequately mitigate stroke risk. Moreover, oral anticoagulant adherence and persistence remain significant challenges in the management of AF. Multiple patient-level factors contribute to poor adherence, including fear of bleeding, low health literacy, emotional distress following diagnosis, and socioeconomic constraints. Factors such as asymptomatic or paroxysmal AF, younger age, limited treatment understanding, and competing life demands further influence medication persistence.

A systematic review of the patient’s clinical condition can provide critical insights to determine the need for in-hospital echocardiography. In addition to AF, our case highlighted important ECG findings such as the presence of crochetage sign, a notching over the R wave apex in the inferior leads, which, in the presence of right axis deviation and partial RBBB, raises strong suspicion of ASD [9,10]. Additional findings that suggested the need for early echocardiography include a loud P2 on auscultation and cardiomegaly evident on chest X-ray. This case emphasizes the importance of a thorough ECG analysis beyond merely confirming AF in stroke patients. When combined with physical signs, this approach supports the prioritization of echocardiography even at the initial stroke presentation. This is crucial as cardioembolic strokes in ASD may occur due to paradoxical embolism or as a result of AF, and both anticoagulation therapy and ASD closure are required to adequately mitigate stroke risk in this patient population.

## Conclusions

While AF remains the major contributor to cardioembolic stroke, careful evaluation of ECG findings and other clinical parameters can help prioritize echocardiographic assessment, particularly for detecting structural heart diseases including acyanotic congenital heart diseases such as ASD. This approach is especially valuable in resource-limited settings, where early echocardiography may not be feasible in all patients with stroke, and prioritization is essential to ensure timely diagnosis and intervention.
